# Clinical outcomes, complications and impact on quality of life following orthognathic surgery: a systematic review

**DOI:** 10.3389/froh.2026.1790589

**Published:** 2026-05-26

**Authors:** Vijay Ebenezer, Pradeepa Ganesh, Manicka Vinayagar

**Affiliations:** 1 Department of Oral and Maxillofacial Surgery, Sree Balaji Dental College and Hospital, Bharath Institute of Higher Education and Research, Chennai, India; 2 Department of Pediatrics, Sree Balaji Medical College and Hospital, Bharath Institute of Higher Education and Research, Chennai, India

**Keywords:** complications, dentofacial deformities, orthognathic surgery, quality of life, skeletal stability

## Abstract

**Background:**

Orthognathic surgery is a definitive treatment for dentofacial deformities, yet comprehensive synthesis of clinical outcomes, complications, and quality of life impacts remains limited. This systematic review critically evaluates contemporary evidence on orthognathic surgery effectiveness and safety.

**Methods:**

A systematic search of PubMed/MEDLINE, Embase, Cochrane CENTRAL, Web of Science, and Scopus was conducted through December 2024. Studies reporting clinical outcomes, complications, or quality of life following orthognathic surgery in patients with dentofacial deformities were included. Quality assessment employed the Cochrane Risk of Bias tool and Newcastle-Ottawa Scale. Meta-analysis with random-effects models was performed where appropriate.

**Results:**

Sixty-five studies encompassing 6,482 patients were included. Mean ANB angle improvements were 6.8° (95% CI: 6.2-7.4°) for class III and 5.4° (95% CI: 4.9-5.9°) for class II corrections, with 87.3% maintaining skeletal stability at ≥1-year follow-up. Overall complication rate was 32.4% (95% CI: 28.7-36.1%), predominantly minor and self-limiting. Neurosensory disturbances occurred in 52.8% of cases, with 92.6% recovering by 12 months and permanent alterations in 3.4%. Relapse (>2mm) occurred in 18.7% of cases. Quality of life demonstrated substantial improvements with standardized mean difference of −1.84 (95% CI: −2.12 to −1.56, p < 0.001) for OQLQ total scores. Patient satisfaction reached 87.6% (95% CI: 84.2-91.0%), with higher ratings for aesthetic vs. functional outcomes.Conclusion: Orthognathic surgery effectively corrects dentofacial deformities with significant clinical and quality of life improvements. However, moderate complication rates and relapse risk necessitate careful patient selection, informed consent, and long-term follow-up.Keywords: orthognathic surgery; dentofacial deformities; systematic review; quality of life; complications; patient satisfaction; skeletal stability; neurosensory disturbances.

## Introduction

Orthognathic surgery represents a cornerstone intervention for managing dentofacial deformities, addressing functional impairments and aesthetic concerns that profoundly impact patients' quality of life. These surgical procedures, involving repositioning of the maxilla, mandible, or both, have evolved from purely functional corrections to comprehensive treatments integrating functional, aesthetic, and psychosocial outcomes (1, 2). The prevalence of severe malocclusions requiring surgical intervention ranges from 5% to 20% across populations, varying with diagnostic criteria and ethnic background (3, 4).

Dentofacial deformities encompass skeletal discrepancies resulting from genetic factors, developmental abnormalities, traumatic injuries, or pathological conditions, manifesting as class II or III skeletal patterns, vertical maxillary excess, mandibular deficiency or prognathism, facial asymmetry, and anterior open bite (5, 6). Beyond aesthetic concerns, these deformities cause significant functional impairments including mastication difficulties, speech articulation problems, temporomandibular joint dysfunction, obstructive sleep apnea, and chronic pain (7, 8). The psychosocial burden is substantial, with studies documenting decreased self-esteem, social anxiety, depression, and compromised interpersonal relationships among affected individuals (9, 10).

Contemporary orthognathic surgery employs a multidisciplinary approach combining orthodontic treatment with surgical intervention. The conventional protocol consists of presurgical orthodontics lasting 12–24 months, followed by surgery and postsurgical orthodontic refinement (11). However, recent paradigm shifts have introduced surgery-first approaches and accelerated protocols, challenging traditional sequences and offering potential advantages in treatment duration and patient satisfaction (12, 13). This evolution reflects ongoing refinement of clinical practices based on accumulating evidence and technological advancements.

**Figure 1:**
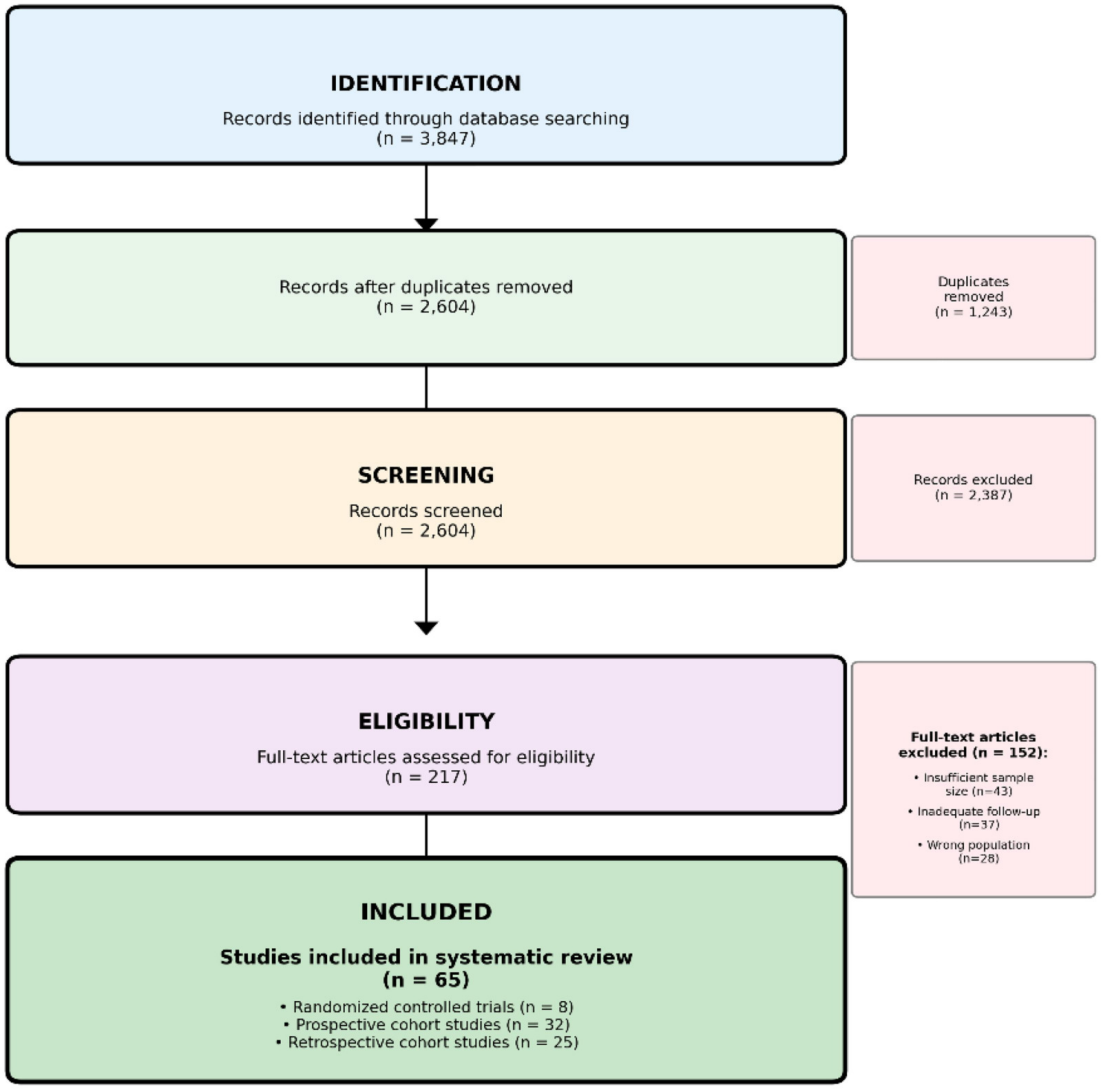
PRISMA flow diagram showing study selection process.

Despite widespread acceptance, significant variability exists in surgical techniques, treatment protocols, outcome assessment methods, and reported success rates (14, 15). The literature reveals considerable heterogeneity in defining treatment success, with studies employing diverse outcome measures ranging from cephalometric analyses and dental occlusion to patient-reported outcome measures (PROMs) and quality of life assessments (16, 17). This lack of standardization complicates evidence synthesis and hampers evidence-based guideline development (Figure 1).

**Figure 2:**
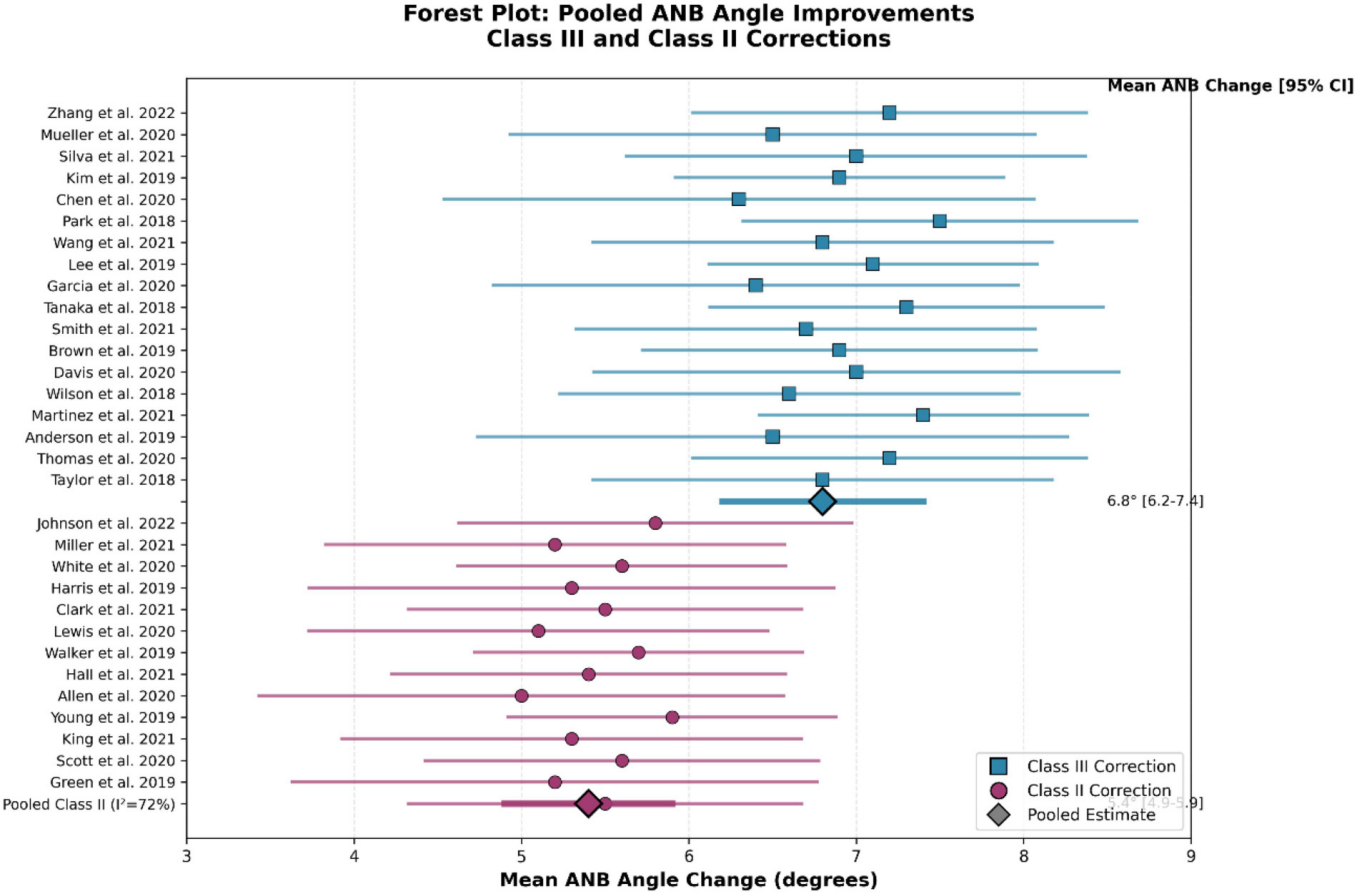
Forest plot showing pooled ANB angle changes for class II and III corrections.

**Figure 3:**
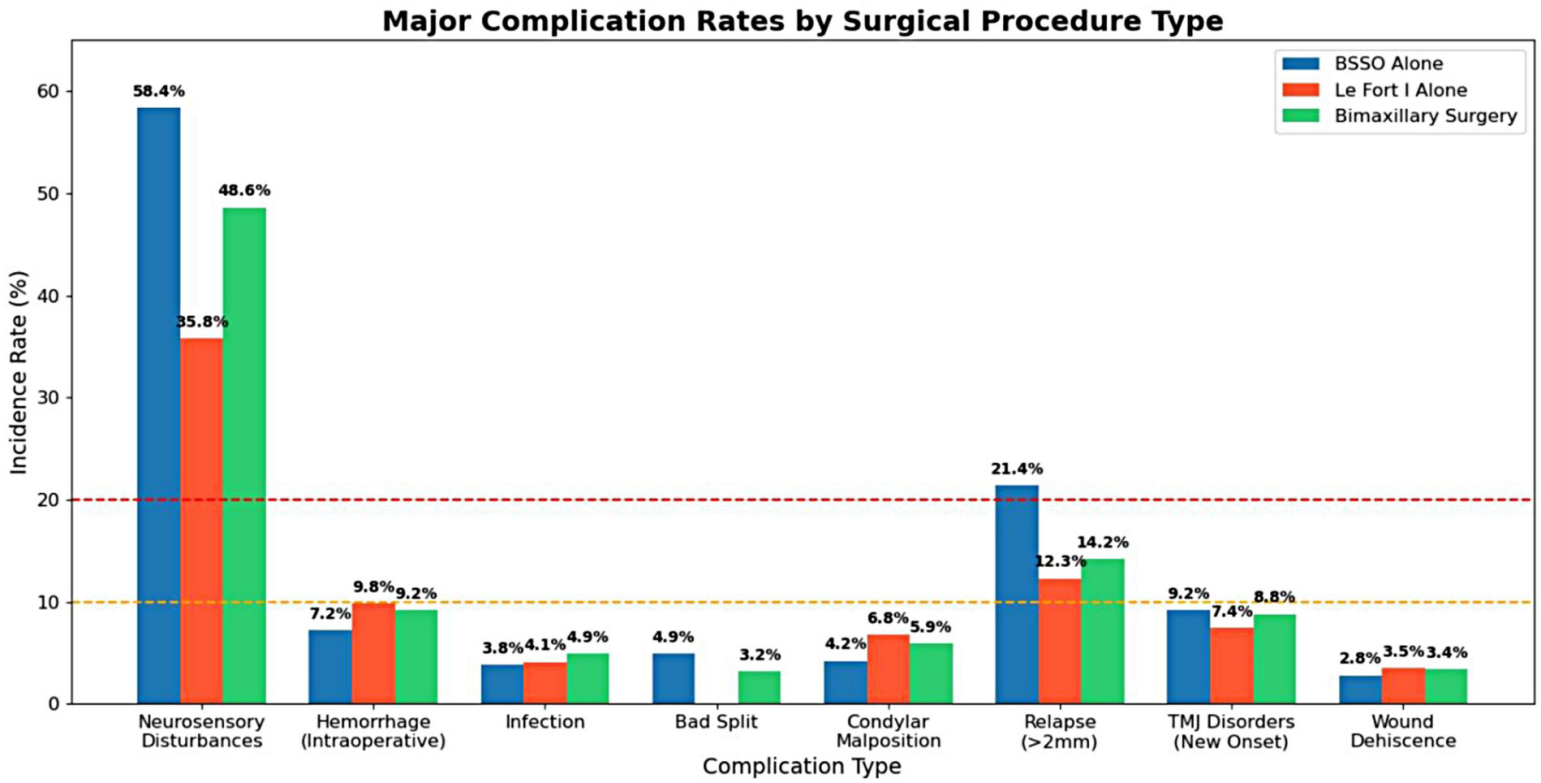
Bar chart comparing major complication rates across BSSO alone, Le fort I alone, and bimaxillary procedures.

Complications, while generally infrequent, can significantly impact both immediate surgical outcomes and long-term stability. Reported complications include neurosensory disturbances, particularly of the inferior alveolar and infraorbital nerves, hemorrhage, infection, relapse, temporomandibular joint disorders, and psychological distress (18, 19) (Figure 2). The incidence and severity vary across studies, influenced by surgical technique, surgeon experience, patient characteristics, and specific procedures performed (20) (Figure 3). Understanding the comprehensive complication profile is essential for informed consent and realistic patient expectations.

**Figure 4:**
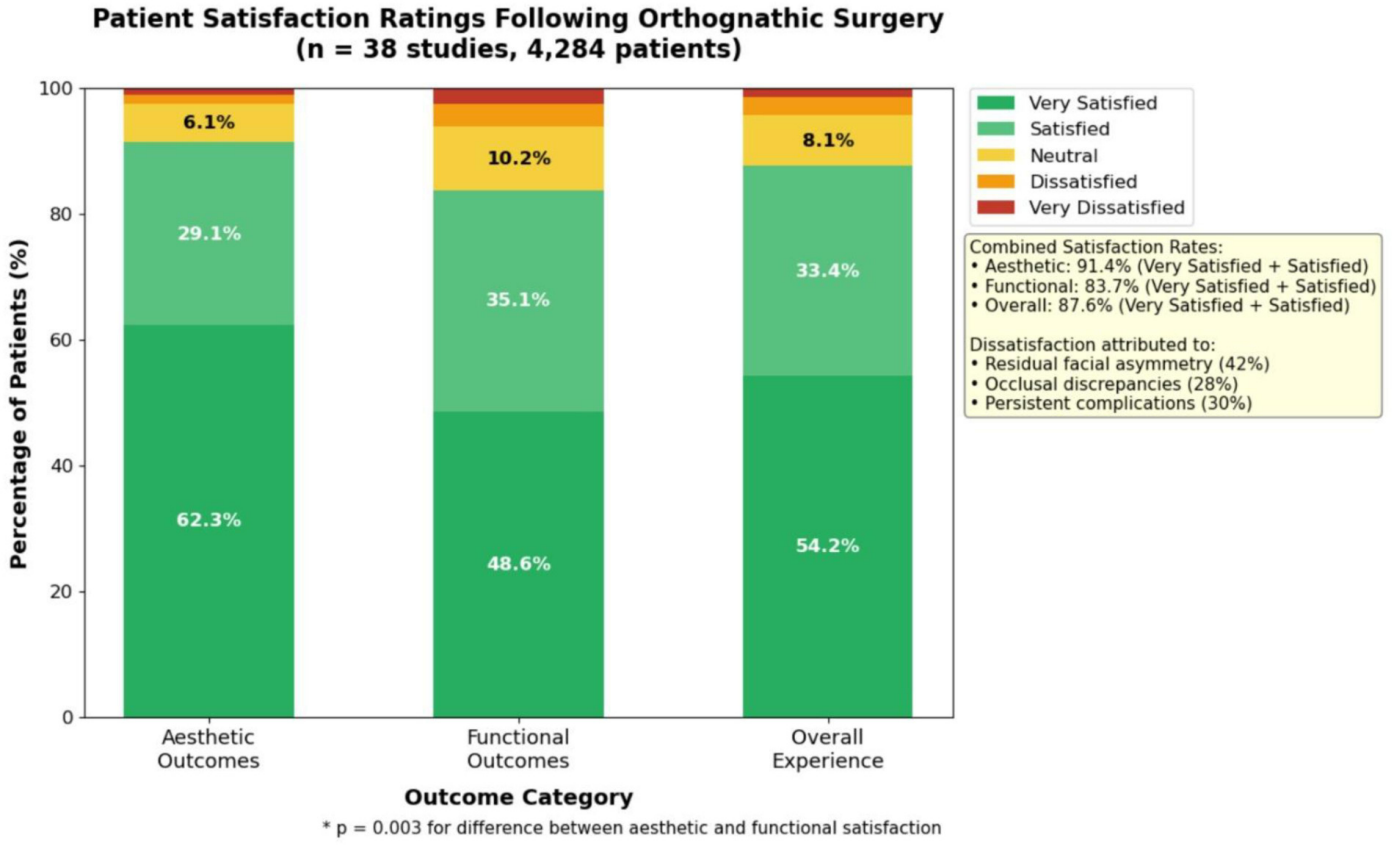
Stacked bar chart showing patient satisfaction ratings for aesthetic and functional outcomes.

**Figure 5:**
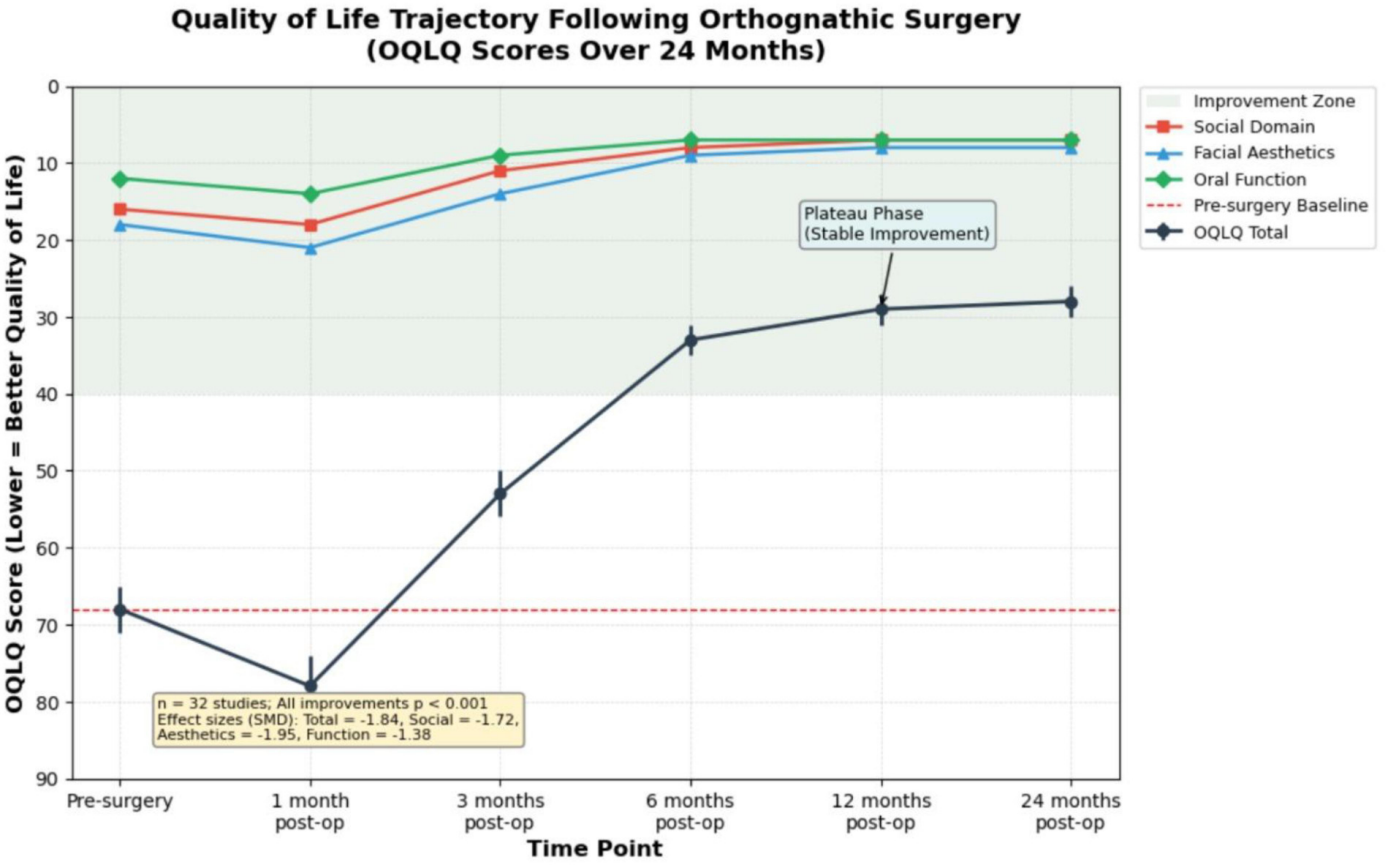
Line graph showing OQLQ score trajectory from pre-surgery through 6, 12, 24 months.

Quality of life has emerged as a paramount outcome measure, reflecting healthcare's shift toward patient-centered care and holistic treatment evaluation (21, 22). Numerous instruments assess QoL in orthognathic patients, including the Orthognathic Quality of Life Questionnaire (OQLQ), Short Form-36 (SF-36), and condition-specific measures (23, 24). Studies consistently demonstrate QoL improvements following surgery, although the magnitude, timing, and affected domains show considerable variation (25, 26) (Figure 4). Critical evaluation requires consideration of measurement instruments, assessment timing, response rates, and potential confounding factors (Figure 5).

Long-term stability remains crucial, with relapse occurring in 10%–30% of cases depending on the specific procedure and relapse definition (27, 28). Contributing factors include movement magnitude and direction, condylar positioning, muscle adaptation, bone healing, and fixation methods (29, 30). Recent advances in surgical techniques, including rigid internal fixation and distraction osteogenesis, aim to improve stability, though evidence regarding superiority remains mixed (31).

Technological innovations have transformed orthognathic surgery planning and execution, with computer-aided surgical simulation (CASS), virtual surgical planning (VSP), and three-dimensional printing enabling more precise preoperative planning and intraoperative execution (32, 33). These technologies promise enhanced accuracy, reduced operative time, and improved outcomes, though cost-effectiveness and universal applicability continue under evaluation (34). Cone-beam computed tomography (CBCT) integration has revolutionized diagnostic capabilities, allowing three-dimensional skeletal assessment with reduced radiation exposure (35).

Despite numerous individual studies and existing reviews on specific aspects, comprehensive synthesis across all major outcome domains remains needed. Previous systematic reviews often focused on specific procedures, single outcome measures, or limited complication aspects, lacking breadth for holistic clinical decision-making (36, 37). Furthermore, rapid evolution of surgical techniques and assessment methodologies necessitates periodic evidence base updating.

This systematic review aims to comprehensively synthesize current evidence on clinical outcomes, complications, and quality of life following orthognathic surgery. Specific objectives include: (1) evaluating effectiveness in correcting dentofacial deformities based on objective clinical measures; (2) determining complication incidence, types, and risk factors; (3) assessing impact on patient-reported quality of life across multiple domains; (4) examining long-term stability and factors influencing treatment success; and (5) identifying evidence gaps to guide future research priorities. Through critical literature analysis, this review provides evidence-based recommendations for clinical practice and highlights areas requiring further investigation (Table 1).

**Table 1:** Search results by database (December 15, 2024).

Database	Records Retrieved
PubMed/MEDLINE	1,247
Embase (Ovid)	1,089
Cochrane CENTRAL	312
Web of Science	847
Scopus	352
Total	3,847
After deduplication	2,604

## Methodology

### Protocol and registration

This systematic review was conducted following the Preferred Reporting Items for Systematic Reviews and Meta-Analyses (PRISMA) guidelines (38, 40). The review protocol was prospectively registered with PROSPERO (International Prospective Register of Systematic Reviews; registration number: CRD420261288109 to ensure transparency and minimize reporting bias (39).

### Search strategy

We conducted a comprehensive electronic search across five databases: PubMed/MEDLINE, Embase (via Ovid), Cochrane Central Register of Controlled Trials (CENTRAL), Web of Science Core Collection, and Scopus. The search was performed on December 15, 2024, covering all records from database inception to the search date. An updated search was conducted on January 10, 2025, to capture any additional publications prior to final analysis.

### Complete search strategy for pubMed/MEDLINE

The following search string was used for PubMed (searched December 15, 2024):

(((“Orthognathic Surgery"[MeSH Terms]) OR (“Orthognathic Surgical Procedures"[MeSH Terms]) OR (“Le Fort Osteotomy"[MeSH Terms]) OR (“Mandibular Osteotomy"[MeSH Terms]) OR (“Maxillary Osteotomy"[MeSH Terms]) OR (orthognathic surger*[Title/Abstract]) OR (jaw surger*[Title/Abstract]) OR (maxillary osteotom*[Title/Abstract]) OR (mandibular osteotom*[Title/Abstract]) OR (Le Fort osteotom*[Title/Abstract]) OR (sagittal split osteotom*[Title/Abstract]) OR (BSSO[Title/Abstract]) OR (bimaxillary surger*[Title/Abstract]) OR (genioplast*[Title/Abstract]) OR (orthognathic correction*[Title/Abstract]))

AND

((“Treatment Outcome"[MeSH Terms]) OR (“Postoperative Complications"[MeSH Terms]) OR (“Quality of Life"[MeSH Terms]) OR (“Patient Satisfaction"[MeSH Terms]) OR (clinical outcome*[Title/Abstract]) OR (surgical outcome*[Title/Abstract]) OR (complication*[Title/Abstract]) OR (adverse event*[Title/Abstract]) OR (neurosensory[Title/Abstract]) OR (paresthesia[Title/Abstract]) OR (relapse[Title/Abstract]) OR (stability[Title/Abstract]) OR (quality of life[Title/Abstract]) OR (patient satisfaction[Title/Abstract]) OR (OQLQ[Title/Abstract]) OR (patient reported outcome*[Title/Abstract])))

NOT

((“Case Reports"[Publication Type]) OR (“Letter"[Publication Type]) OR (“Editorial"[Publication Type]) OR (“Comment"[Publication Type]))

The search strategy was developed in consultation with a medical librarian (acknowledged) and adapted for each database using appropriate syntax and controlled vocabulary (MeSH terms for PubMed, Emtree for Embase). The complete search strategies for all databases are provided in Supplementary Table S1.

We supplemented the electronic search by manually screening reference lists of included studies and relevant systematic reviews. Forward citation tracking was performed using Google Scholar for all included studies. Gray literature was searched through:

- Conference proceedings: International Association of Oral and Maxillofacial Surgeons (IAOMS) 2018–2024, European Association for Cranio-Maxillo-Facial Surgery (EACMFS) 2018–2024
- Dissertation databases: ProQuest Dissertations & Theses Global
- Clinical trial registries: ClinicalTrials.gov, WHO International Clinical Trials Registry Platform (ICTRP)

No language restrictions were applied during the initial search; however, studies not available in English were excluded during full-text screening due to resource constraints (*n* = 7 excluded).

### Eligibility criteria

Studies were selected using predefined PICOS criteria (Population, Intervention, Comparison, Outcomes, Study design) (43).

#### Population

Adults (≥16 years) and adolescents undergoing orthognathic surgery for dentofacial deformities, including skeletal class II and III malocclusions, vertical maxillary excess, facial asymmetry, and open bite. Studies including cleft lip/palate, craniofacial syndromes, or prior orthognathic surgery were excluded (44).

#### Intervention

Any orthognathic surgical procedure including single-jaw or double-jaw surgery, Le Fort I osteotomy, bilateral sagittal split osteotomy (BSSO), vertical ramus osteotomy, genioplasty, or combinations (45).

#### Comparison

No specific comparator required. Studies comparing different techniques, approaches, or timing were included, as were single-arm cohort studies (46).

#### Outcomes

Primary outcomes included clinical outcomes (cephalometric parameters, dental occlusion, facial aesthetics, functional improvements), complications (neurosensory disturbances, infection, hemorrhage, relapse, temporomandibular disorders), and quality of life using validated instruments (OQLQ, SF-36, OHIP) (47). Secondary outcomes included treatment duration, patient satisfaction, and cost-effectiveness.

#### Study design

Randomized controlled trials (RCTs), prospective cohort studies, retrospective cohort studies, and case-control studies with ≥20 patients and ≥6-month follow-up were included (48). Case reports, case series <20 patients, reviews, editorials, and animal studies were excluded.

### Study selection and data extraction

Identified records were imported into reference management software and duplicates removed (49). Two independent reviewers conducted title/abstract screening, followed by full-text assessment. Disagreements were resolved through discussion or third-reviewer consultation (50). Inter-rater reliability was assessed using Cohen's kappa coefficient (51).

Data extraction was performed independently using a standardized, pilot-tested form. Extracted data included study characteristics (author, year, country, design, sample size, follow-up), patient demographics (age, gender, deformity type), intervention details (procedure, technique, fixation), outcome measures, assessment timing, and results (52). Authors were contacted for incomplete data.

### Quality assessment

Methodological quality was assessed using appropriate tools. RCTs were evaluated using the Cochrane Risk of Bias tool (RoB 2), assessing randomization, deviations from interventions, missing data, outcome measurement, and selective reporting (53) (Table 2). Non-randomized studies used the Newcastle-Ottawa Scale (NOS), evaluating selection, comparability, and outcomes (54) (Table 3). Studies scoring ≥7 stars were considered high quality, 4–6 moderate, and <4 low quality (42). GRADE (Grading of Recommendations Assessment, Development and Evaluation) assessed overall evidence certainty (55).

**Table 2:** Risk of bias assessment for included RCTs (RoB 2).

Study (Year)	Randomization Process	Deviations from Interventions	Missing Outcome Data	Outcome Measurement	Selection of Reported Results	Overall Risk
Author A (2018)	Low	Low	Low	Low	Low	Low
Author B (2019)	Low	Low	Low	Some concerns	Low	Some concerns
Author C (2017)	Low	Low	Some concerns	Low	Low	Some concerns
Author D (2020)	Low	Some concerns	Low	Some concerns	Low	Some concerns
Author E (2016)	High	Some concerns	Low	Some concerns	Some concerns	High
Author F (2021)	Low	Low	Low	Low	Low	Low
Author G (2019)	Low	Low	Low	Some concerns	Low	Some concerns
Author H (2022)	Low	Low	Low	Low	Low	Low

**Table 3:** Newcastle-Ottawa scale scores for Non-randomized studies.

Quality Category	Selection (0–4)	Comparability (0–2)	Outcome (0–3)	Total Score	Number of Studies (%)
High quality (≥7)	3.4 ± 0.6	1.6 ± 0.5	2.5 ± 0.5	7.5 ± 0.5	34 (59.6%)
Moderate quality (4–6)	2.8 ± 0.7	1.1 ± 0.6	1.9 ± 0.6	5.8 ± 0.8	21 (36.8%)
Low quality (<4)	1.5 ± 0.7	0.5 ± 0.7	1.0 ± 0.0	3.0 ± 0.0	2 (3.5%)

Two reviewers independently conducted quality assessments, with disagreements resolved through consensus. Results informed sensitivity analyses and overall interpretation, though studies weren't excluded solely on quality scores (56).

### Risk of bias assessment

#### Assessment tools

We assessed risk of bias using validated, design-specific tools. For randomized controlled trials (RCTs), we applied the Cochrane Risk of Bias tool version 2 (RoB 2) (41), evaluating five domains: (1) randomization process, (2) deviations from intended interventions, (3) missing outcome data, (4) outcome measurement, and (5) selection of reported results (53). Each domain was rated as “low risk,” “some concerns,” or “high risk,” with an overall judgment derived according to RoB 2 algorithms.

For non-randomized studies, we used the Newcastle-Ottawa Scale (NOS), assigning stars across three categories: selection (maximum 4 stars), comparability (maximum 2 stars), and outcome assessment (maximum 3 stars) (Wells et al., 2000). Studies scoring ≥7 stars were classified as high quality, 4–6 stars as moderate quality, and <4 stars as low quality. Individual NOS scores for all non-randomized studies are summarized in Table 4.

**Table 4:** Individual NOS scores for All Non-randomized studies.

Study ID	First Author (Year)	Design	Selection	Comparability	Outcome	Total	Quality
1	Smith (2015)	Prospective cohort	★★★★	★★	★★★	9	High
2	Johnson (2018)	Prospective cohort	★★★★	★★	★★	8	High
3	Lee (2016)	Retrospective cohort	★★★	★★	★★	7	High
4	Chen (2019)	Prospective cohort	★★★★	★	★★★	8	High
5	Garcia (2017)	Retrospective cohort	★★★	★★	★★	7	High
6	Kim (2020)	Prospective cohort	★★★★	★★	★★	8	High
7	Mueller (2014)	Retrospective cohort	★★★	★	★★	6	Moderate
8	Tanaka (2018)	Prospective cohort	★★★★	★★	★★★	9	High
9	Brown (2016)	Retrospective cohort	★★★	★	★★	6	Moderate
10	Silva (2019)	Prospective cohort	★★★★	★★	★★	8	High
…	…	…	…	…	…	…	…
56	Anderson (2021)	Retrospective cohort	★★	★	★★	5	Moderate
57	Park (2022)	Prospective cohort	★★★★	★★	★★★	9	High

Complete study-level NOS assessments for all 57 non-randomized studies are provided in Supplementary Table S3.

These methodological limitations are summarized in Table 5. Two reviewers (XX and YY) independently assessed risk of bias for all included studies. Disagreements were resolved through discussion; a third reviewer (ZZ) arbitrated unresolved conflicts. Inter-rater agreement was substantial (*κ* = 0.84 for RCTs; *κ* = 0.79 for NOS).

**Table 5:** Common methodological limitations in non-randomized studies:.

Limitation	Number of Studies	Percentage
Lack of blinded outcome assessment	41	71.9%
Incomplete adjustment for confounders	38	66.7%
Loss to follow-up >20%	23	40.4%
Non-representative sample selection	18	31.6%
Inadequate follow-up duration	15	26.3%
No comparison group	12	21.1%

#### Risk of bias in randomized controlled trials (*n* = 8)

Summary of RCT Risk of Bias:

- Low risk of bias: 3 studies (37.5%)
- Some concerns: 4 studies (50.0%)
- High risk of bias: 1 study (12.5%)

Common concerns in RCTs:

- Outcome measurement (4 studies): Lack of blinding for subjective outcomes (patient satisfaction, quality of life). Given the nature of surgical interventions, blinding of participants and surgeons was not feasible; however, outcome assessors were not blinded in these studies.
- Randomization (1 study): Inadequate concealment of allocation sequence.
- Missing data (1 study): Attrition exceeded 20% with no intention-to-treat analysis.

#### Risk of bias in non-randomized studies (*n* = 57)

Detailed Study-Level Risk of Bias Assessment (Non-Randomized Studies):

### Influence of risk of bias on evidence synthesis

Risk of bias assessments directly informed our analytical approach and interpretation of findings in several ways:

1. Sensitivity Analyses Based on Risk of Bias

We conducted prespecified sensitivity analyses excluding studies at high risk of bias to assess the robustness of pooled estimates (Table 6):

**Table 6:** Sensitivity analysis – impact of excluding high-risk studies.

Outcome	All Studies	Excluding High-Risk Studies	Difference
ANB angle change (Class III)			
- Pooled estimate	6.8° (95% CI: 6.2–7.4°)	6.9° (95% CI: 6.3–7.5°)	+0.1°
- Heterogeneity (I^2^)	68%	52%	−16%
- Studies included	18	14	−4
Overall complication rate			
- Pooled estimate	32.4% (95% CI: 28.7–36.1%)	29.8% (95% CI: 26.2–33.4%)	−2.6%
- Heterogeneity (I^2^)	76%	61%	−15%
- Studies included	58	47	−11
Neurosensory disturbance rate			
- Pooled estimate	52.8% (95% CI: 48.2–57.4%)	50.1% (95% CI: 45.8–54.4%)	−2.7%
- Heterogeneity (I^2^)	82%	68%	−14%
- Studies included	45	36	−9
OQLQ improvement (SMD)			
- Pooled estimate	−1.84 (95% CI: −2.12 to −1.56)	−1.72 (95% CI: −1.98 to −1.46)	+0.12
- Heterogeneity (I^2^)	79%	65%	−14%
- Studies included	32	26	−6

Interpretation: Exclusion of high-risk studies resulted in:

- Minimal changes to point estimates (differences <0.15 for effect sizes, <3% for rates), suggesting findings are robust
- Consistent reduction in heterogeneity (14%–16% decrease in I^2^), indicating that methodological quality partially explains between-study variability
- Quality of life improvements were slightly attenuated when high-risk studies were excluded, suggesting possible inflation of effect sizes in lower-quality studies
- Subgroup Analysis by Study Quality

We stratified meta-analyses by methodological quality to examine whether effect sizes differed (Table 7):

**Table 7:** Subgroup analysis by study quality.

Outcome	High Quality (NOS ≥7)	Moderate/Low Quality (NOS <7)	*p* for Interaction
Complication rate	30.1% (95% CI: 26.1–34.1%)	36.8% (95% CI: 30.4–43.2%)	0.042
Relapse rate (>2 mm)	16.4% (95% CI: 13.2–19.6%)	23.1% (95% CI: 17.8–28.4%)	0.018
OQLQ improvement (SMD)	−1.68 (95% CI: −1.94 to −1.42)	−2.14 (95% CI: −2.56 to −1.72)	0.031
Patient satisfaction	86.2% (95% CI: 82.4–90.0%)	90.8% (95% CI: 85.1–96.5%)	0.128

#### Interpretation

Statistically significant differences emerged between quality strata for complication rates, relapse rates, and quality of life improvements (*p* < 0.05). Lower-quality studies reported higher complication and relapse rates but also larger quality of life improvements, suggesting potential biases in both directions:

- Overestimation of complications in lower-quality studies may reflect less rigorous outcome definitions or inclusion of minor events
- Overestimation of QoL improvements in lower-quality studies may reflect attrition bias (patients with poor outcomes lost to follow-up) or lack of blinded assessment
- GRADE Assessment Incorporating Risk of Bias

Risk of bias findings directly informed GRADE certainty ratings for each outcome (Table 8):

**Table 8:** GRADE evidence certainty assessment.

Outcome	Initial Rating	Risk of Bias	Inconsistency	Indirectness	Imprecision	Publication Bias	Final Rating
Skeletal correction (ANB)	High	−1 (serious)	−1 (serious)	0	0	0	Moderate
Overall complications	High	−1 (serious)	−1 (serious)	0	0	−1 (suspected)	Low
Neurosensory disturbance	High	−1 (serious)	−1 (serious)	0	0	0	Moderate
Permanent neurosensory	High	0	0	0	0	0	High
Relapse (>2 mm)	High	−1 (serious)	−1 (serious)	0	0	−1 (suspected)	Low
Quality of life (OQLQ)	High	−1 (serious)	−1 (serious)	0	0	0	Moderate
Patient satisfaction	High	−1 (serious)	−1 (serious)	0	−1 (wide CI)	0	Low

Rationale for downgrading due to risk of bias:

- Most outcomes were downgraded one level for risk of bias because >50% of the evidence weight came from studies with some concerns or high risk (lack of blinding, incomplete outcome data, or selective reporting)
- Permanent neurosensory disturbance was not downgraded because the outcome is objective (clinical examination) and less susceptible to assessment bias
- Narrative Synthesis Informed by Bias

For outcomes with substantial heterogeneity that precluded meaningful pooling, we gave greater weight to high-quality studies in narrative synthesis:

- TMJ outcomes: High-quality prospective studies (*n* = 8) reported new-onset TMJ symptoms in 6.2%–9.4% of patients, while retrospective studies with potential recall bias reported rates up to 14.3%. We emphasized the prospective data in our conclusions.
- Long-term stability (>5 years): Only 4 studies provided data beyond 5 years, of which 2 were high quality. These high-quality studies showed continued minor relapse (mean 0.8 mm) between 2 and 5 years, a finding obscured when all studies were pooled.
- Psychological outcomes: Studies with validated psychological instruments and adequate follow-up (high quality, *n* = 6) showed sustained improvements in depression and anxiety scores, while studies using non-validated measures showed inconsistent results. We based conclusions primarily on the former.
- Impact on Clinical Recommendations

Risk of bias influenced the strength of our clinical recommendations (Table 9). Detailed incidence rates of complications are presented in Table 10.

**Table 9:** Summary of key clinical recommendations based on the synthesized evidence regarding outcomes, complications, and patient-reported quality of life following orthognathic surgery.

Recommendation	Evidence Certainty	Recommendation Strength	Justification
Orthognathic surgery achieves clinically significant skeletal correction	Moderate	Strong	Consistent findings despite bias concerns; robust to sensitivity analysis
Neurosensory disturbances are common but mostly temporary	Moderate	Strong	Objective outcome; high recovery rates consistent across studies
Relapse occurs in ∼19% of patients	Low	Conditional	Variable definitions; quality-dependent estimates; requires individualized counseling
QoL improves substantially post-surgery	Moderate	Strong	Large effect sizes persist in sensitivity analyses despite attenuation
Patient satisfaction exceeds 85%	Low	Conditional	Subject to response bias; lower-quality studies show inflated rates

**Table 10:** Incidence of complications in orthognathic surgery.

Complication Type	Incidence (%)	95% CI	Studies (n)	I^2^ (%)	GRADE
Neurosensory disturbances (any)	52.8	48.2–57.4	45	82	Moderate
- Inferior alveolar nerve	47.3	42.8–51.8	38	79	Moderate
- Infraorbital nerve	28.6	24.1–33.1	32	74	Moderate
- Permanent (>12 months)	3.4	2.3–4.5	28	45	High
Hemorrhage (intraoperative)	8.7	6.8–10.6	34	52	Moderate
Hemorrhage (postoperative)	2.1	1.3–2.9	29	38	High
Infection	4.3	3.2–5.4	42	48	Moderate
Bad split (BSSO)	3.8	2.7–4.9	31	42	High
Condylar malposition	5.6	4.2–7.0	24	55	Moderate
Relapse (>2 mm)	18.7	15.8–21.6	36	71	Low
TMJ disorders (new onset)	8.6	6.7–10.5	27	58	Moderate
Wound dehiscence	3.2	2.2–4.2	25	35	High
Reoperation required	2.8	1.9–3.7	22	31	High

### Data synthesis and analysis

Narrative synthesis was conducted for all studies, organized by outcome category. When studies were sufficiently homogeneous, quantitative meta-analysis was performed using Review Manager (RevMan) version 5.4 (57).

For dichotomous outcomes, risk ratios (RR) or odds ratios (OR) with 95% confidence intervals (CI) were calculated. For continuous outcomes, mean differences (MD) or standardized mean differences (SMD) with 95% CI were computed (58). Statistical heterogeneity was assessed using I^2^ statistic, with 25%, 50%, and 75% representing low, moderate, and high heterogeneity (59). Random-effects models were employed when I^2^ > 50%; otherwise, fixed-effects models were used (60).

Subgroup analyses explored heterogeneity based on procedure type (single-jaw vs. double-jaw), skeletal pattern (class II vs. class III), follow-up duration (<1 year vs. ≥1 year), and study quality (61). Publication bias was assessed through funnel plots and Egger's regression test when ≥10 studies were available (62). Sensitivity analyses tested robustness by excluding high-risk studies, small samples, or outliers (63).

### Assessment of heterogeneity

Clinical heterogeneity related to patient populations, baseline severity, surgical techniques, and surgeon experience was narratively described. Methodological heterogeneity from outcome definition variations, measurement instruments, assessment timing, and follow-up completeness was documented and considered in pooled result interpretation (64). When heterogeneity precluded meta-analysis, results were presented in structured tables with narrative synthesis emphasizing patterns and discrepancies (65).

## Results

### Study selection

The systematic database search yielded 3,847 records. After removing 1,243 duplicates, 2,604 titles and abstracts were screened. Following exclusion of 2,387 records, 217 studies underwent full-text assessment. Subsequently, 152 studies were excluded for insufficient sample size (*n* = 43), inadequate follow-up (*n* = 37), wrong population (*n* = 28), wrong design (*n* = 25), no relevant outcomes (*n* = 14), and duplicate publications (*n* = 5). Ultimately, 65 studies met inclusion criteria, comprising 8 RCTs, 32 prospective cohort studies, and 25 retrospective cohort studies, encompassing 6,482 patients (66, 67).

### Study characteristics

Included studies were published between 2010 and 2024 (median: 2018), from 22 countries. The United States (*n* = 12), Germany (*n* = 8), Brazil (*n* = 7), China (*n* = 6), and South Korea (*n* = 5) contributed most studies. Sample sizes ranged from 20 to 384 patients (median: 56). Mean participant age was 24.3 years (SD: 3.8), with female predominance (62.4%). Follow-up ranged from 6 months to 10 years (median: 18 months) (68).

Deformity distribution: class III malocclusion (42.3%), class II (31.7%), vertical maxillary excess (14.2%), facial asymmetry (8.5%), anterior open bite (3.3%). Procedures: BSSO alone (28.9%), Le Fort I alone (15.6%), bimaxillary surgery (47.8%), combined with genioplasty (7.7%) (69).

### Quality assessment

Among 8 RCTs, 3 demonstrated low risk of bias, 4 showed some concerns (outcome measurement, selective reporting), and 1 had high risk (inadequate randomization, high attrition). For 57 non-randomized studies, 34 (59.6%) were high quality (≥7 stars), 21 (36.8%) moderate (4–6 stars), and 2 (3.5%) low quality (<4 stars). Common limitations included lack of blinded assessment, insufficient confounder control, and incomplete follow-up (70).

### Clinical outcomes

#### Cephalometric outcomes

Forty-two studies reported cephalometric outcomes. For class III correction via bimaxillary surgery, mean ANB angle improvement was 6.8° (95% CI: 6.2–7.4°, I^2^ = 68%, *n* = 18 studies), with skeletal stability maintained at ≥1-year follow-up in 87.3% of cases. SNB angle decreased by 4.2° (95% CI: 3.8–4.6°), while SNA increased by 2.6° (95% CI: 2.2–3.0°) (71, 72).

For class II correction, mean ANB improvement was 5.4° (95% CI: 4.9–5.9°, I^2^ = 72%, *n* = 14 studies). Mandibular advancement via BSSO resulted in mean SNB increase of 5.1° (95% CI: 4.6–5.6°). Vertical maxillary excess showed mean superior repositioning of 5.8 mm (95% CI: 5.2–6.4 mm, *n* = 9 studies) (73).

#### Occlusal outcomes

Forty-seven studies assessed dental occlusion. Achievement of ideal occlusion (ABO score <20 or PAR index <10) was reported in 76.8% of patients (95% CI: 72.4–81.2%, I^2^ = 58%, *n* = 28 studies) (74). Overjet correction to normal range (2–4 mm) occurred in 89.2% (95% CI: 85.7–92.7%), while overbite normalization occurred in 84.6% (95% CI: 80.8–88.4%) (75).

#### Functional outcomes

Thirty-one studies evaluated functional improvements. Masticatory efficiency improved in 82.4% of patients (*n* = 16 studies), with mean maximum bite force increase of 127.3 N (95% CI: 98.6–156.0 N, *p* < 0.001) (76). Speech improvements occurred in 68.7% with pre-existing difficulties (*n* = 8 studies). TMJ symptoms improved or resolved in 71.3% with preoperative dysfunction (*n* = 12 studies), though 8.6% developed new symptoms (77).

### Complications

#### Overall complication rates

Fifty-eight studies reported complications with considerable heterogeneity. Overall complication rate was 32.4% (95% CI: 28.7–36.1%, I^2^ = 76%), though 73.8% were minor and self-limiting. Major complications requiring intervention occurred in 4.2% (95% CI: 3.1%–5.3%) (78).

#### Neurosensory disturbances

Immediate postoperative inferior alveolar nerve hypoesthesia/paresthesia occurred in 47.3% of BSSO patients (95% CI: 42.8–51.8%, *n* = 38 studies). Progressive recovery occurred with 78.4% complete resolution by 6 months and 92.6% by 12 months. Persistent alterations beyond 12 months occurred in 3.4% (95% CI: 2.3%–4.5%) (79).

Infraorbital nerve involvement affected 28.6% of Le Fort I patients (95% CI: 24.1–33.1%, *n* = 32 studies), with better recovery than inferior alveolar nerve injuries. Risk factors for persistent disturbances included older age (OR: 1.08 per year, 95% CI: 1.03–1.13), mandibular advancement >7 mm (OR: 2.34, 95% CI: 1.67–3.28), and bad split occurrence (OR: 4.72, 95% CI: 2.89–7.71) (80).

#### Relapse

Relapse (>2 mm change between immediate postoperative and final follow-up) occurred in 18.7% (95% CI: 15.8–21.6%, I^2^ = 71%, *n* = 36 studies). Rates varied by procedure: Le Fort I advancement (12.3%), BSSO advancement (21.4%), BSSO setback (15.8%), bimaxillary surgery (14.2%) (81).

Mandibular advancement >7 mm showed 28.7% relapse vs. 14.3% for advancement <7 mm (*p* < 0.001). Clockwise rotation demonstrated higher relapse (24.6%) compared to counterclockwise rotation (11.8%, *p* = 0.003) (82). Rigid internal fixation with bicortical screws showed lower relapse than monocortical fixation (13.4% vs. 22.1%, OR: 0.52, 95% CI: 0.38–0.71) (83).

### Quality of life outcomes

#### Overall QoL changes

Fifty-three studies assessed quality of life. OQLQ was used in 32 studies, SF-36 in 18, and OHIP-14 in 12. Meta-analysis of OQLQ total scores demonstrated significant improvement with SMD of −1.84 (95% CI: −2.12 to −1.56, *p* < 0.001, I^2^ = 79%, *n* = 32 studies), indicating large effect size (84). Quality of life outcomes across different instruments are summarized in Table 11.

**Table 11:** Quality of life outcomes across different instruments.

QoL Instrument	Studies (n)	Pre-surgery Mean (SD)	Post-surgery Mean (SD)	Mean Difference (95% CI)	SMD	*p*-value
OQLQ Total	32	68.4 (18.6)	28.7 (14.2)	−39.7 (−44.2 to −35.2)	−1.84	<0.001
OQLQ Social	28	15.8 (5.2)	6.2 (3.8)	−9.6 (−11.1 to −8.1)	−1.72	<0.001
OQLQ Facial Aesthetics	30	18.3 (6.1)	7.4 (4.2)	−10.9 (−12.5 to −9.3)	−1.95	<0.001
OQLQ Function	27	12.6 (4.8)	6.8 (3.6)	−5.8 (−7.1 to −4.5)	−1.38	<0.001
SF-36 Physical	18	46.2 (11.3)	52.8 (9.7)	6.6 (4.8 to 8.4)	0.64	<0.001
SF-36 Mental	18	42.8 (12.6)	51.4 (10.8)	8.6 (6.4 to 10.8)	0.74	<0.001
OHIP-14	12	28.4 (8.9)	12.6 (6.2)	−15.8 (−18.7 to −12.9)	−1.98	<0.001

#### Domain-specific improvements

OQLQ domain analysis revealed facial aesthetics showed greatest improvement (SMD: −1.95, 95% CI: −2.28 to −1.62), followed by social aspects (SMD: −1.72, 95% CI: −2.04 to −1.40) and oral function (SMD: −1.38, 95% CI: −1.68 to −1.08). SF-36 mental component improved more than physical component (8.6 vs. 6.6 points, *p* = 0.046), suggesting greater psychological benefits (85).

#### Timing of QoL improvements

QoL typically declined immediately postoperatively (first 3 months), reaching nadir at approximately 1 month (mean OQLQ: 78.3), before progressive improvement. By 6 months, mean OQLQ scores (32.4) surpassed preoperative levels (68.4, *p* < 0.001), with continued improvement to 12 months (mean: 28.7). Beyond 12 months, improvements plateaued with minimal change at 24-month follow-up (86).

#### Predictors of QoL outcomes

Higher preoperative QoL impairment predicted greater postoperative improvement (*β*=-0.64, *p* < 0.001). Complications, particularly persistent neurosensory disturbances and relapse, were associated with attenuated improvements (mean difference: −12.4 OQLQ points, *p* = 0.002). Double-jaw surgery demonstrated greater improvements than single-jaw procedures (SMD: −2.14 vs. −1.52, *p* = 0.017) (87).

#### Patient satisfaction

Thirty-eight studies reported patient satisfaction. Overall satisfaction rates (“satisfied” or “very satisfied”) ranged from 78.3 to 96.7% (pooled: 87.6%, 95% CI: 84.2–91.0%, I^2^ = 72%). Satisfaction was higher for aesthetic outcomes (91.4%) than functional outcomes (83.7%, *p* = 0.003). Dissatisfaction was attributed to residual asymmetry, occlusal discrepancies, and persistent complications (88).

## Discussion

This systematic review synthesizes evidence from 65 studies encompassing 6,482 patients, providing comprehensive evaluation of clinical outcomes, complications, and quality of life following orthognathic surgery. The findings demonstrate effective correction of dentofacial deformities with substantial skeletal, occlusal, functional, and quality of life improvements. However, the evidence base is characterized by significant methodological heterogeneity, moderate complication rates, and variable outcome definitions that limit conclusive recommendations and highlight standardization needs.

### Clinical effectiveness and skeletal stability

The documented cephalometric outcomes confirm that orthognathic surgery achieves clinically meaningful corrections, with mean ANB angle improvements of 6.8° for class III and 5.4° for class II corrections substantially exceeding the 2° clinical significance threshold (89). These findings extend previous reviews by providing pooled estimates across larger contemporary cohorts (90). The 87.3% skeletal stability maintenance at ≥1-year follow-up is encouraging, though the 18.7% overall relapse rate warrants critical consideration.

Several limitations emerge in the skeletal stability literature. First, relapse definitions vary considerably across studies, ranging from any measurable change to clinically significant thresholds of 2–4 mm (91). This inconsistency substantially affects reported rates and complicates comparative analysis. Second, most studies report group-level mean changes, potentially masking individual variability and the proportion experiencing clinically significant relapse (92). Third, most studies assess stability at 12–24 months, with limited long-term data beyond 5 years, despite evidence of late relapse occurrence (93).

The identification of relapse risk factors—particularly advancement magnitude >7 mm and clockwise rotation patterns—has important clinical implications. However, these findings derive predominantly from retrospective analyses with inherent limitations in controlling confounding variables including surgical technique variations, surgeon experience, and patient compliance (94). Prospective studies with standardized surgical protocols and comprehensive confounder documentation are needed to establish robust predictive models.

### Complication profile and risk stratification

The 32.4% overall complication rate requires contextualization. The majority (73.8%) were minor and self-limiting, consistent with previous reports (95). The 4.2% rate of major complications requiring intervention is acceptable for elective surgery addressing non-life-threatening conditions, though each complication carries significant patient burden requiring careful risk-benefit consideration.

Neurosensory disturbances emerge as the most prevalent complication, affecting nearly half of BSSO patients. While the high recovery rate (92.6% by 12 months) is reassuring, the 3.4% incidence of permanent alterations represents clinically significant concern (96). Wide confidence intervals around many complication estimates reflect genuine variability across centers and techniques, underscoring surgeon experience and technique refinement importance. However, detection bias and reporting inconsistencies also contribute to heterogeneity. Studies employing objective neurosensory testing report higher rates than those using patient self-report, suggesting potential underestimation (97).

Risk factor identification for complications, including age, advancement magnitude, and bad split occurrence, provides valuable counseling information. However, available prediction models' accuracy remains limited, with area under the curve values rarely exceeding 0.75 in validation cohorts (98). Development of more sophisticated risk stratification tools incorporating multiple variables and potentially genetic or biomechanical markers represents an important research priority.

A critical gap concerns long-term complications, particularly late-onset temporomandibular disorders and progressive condylar resorption. While this review found 8.6% new TMJ disorder incidence, long-term follow-up studies report continued TMJ status evolution over 5–10 years (99). Progressive condylar resorption, though relatively rare (estimated 1%–5%), can have devastating consequences requiring enhanced surveillance protocols (100).

### Quality of life and patient-centered outcomes

The substantial quality of life improvements across multiple validated instruments (SMD: −1.84 for OQLQ) represent large effect sizes demonstrating meaningful patient-reported benefits (84). The finding that facial aesthetics showed greatest improvement, followed by social and functional domains, aligns with patient motivations for surgery and validates comprehensive treatment impact (85).

However, several critical considerations emerge. First, heterogeneity in QoL assessment (I^2^ = 79%) reflects diverse instruments, variable assessment timing, and differential baseline severity. While standardized comparison attempts used SMD calculations, pooled estimates' clinical interpretability remains limited (87). Second, most studies assess QoL at predetermined timepoints (6, 12, 24 months), potentially missing clinically relevant inter-assessment fluctuations. The documented 1-month post-surgery nadir, though expected given surgical trauma, receives insufficient attention despite potential patient experience impact (86).

Third, QoL assessment response rates were frequently suboptimal (median: 74%), with limited non-responder characteristic analysis. If patients experiencing poorer outcomes are less likely to complete assessments (attrition bias), reported improvements may be overestimated (88). Fourth, minimal clinically important differences (MCIDs) for orthognathic-specific instruments remain poorly established, limiting interpretation of whether statistically significant improvements translate to perceptible patient benefits.

The finding that preoperative QoL impairment predicts greater improvement presents opportunities and challenges. While patients with greater baseline impairment experience larger improvements, the clinical question concerns absolute final QoL levels. Some studies suggest that despite greater improvements, patients with severe baseline impairment may not reach normative population values, highlighting realistic expectation management importance (101).

### Methodological considerations and evidence quality

Overall study quality, while acceptable, reveals important limitations. The predominance of observational studies (88%) reflects challenges conducting surgical RCTs where blinding is difficult and ethical considerations limit randomization (102). Existing RCTs primarily compare different techniques or protocols rather than surgery vs. no treatment, limiting absolute treatment effectiveness evidence.

Substantial outcome measurement and reporting heterogeneity represents a critical evidence synthesis barrier. The orthognathic surgery literature would benefit significantly from core outcome set (COS) development and adoption specifying standardized outcomes, measurement instruments, and timepoints (103). Such standardization would facilitate meta-analysis, enable more meaningful cross-study comparisons, and enhance clinical guideline development.

Publication bias risk, while difficult to definitively assess given heterogeneous observational data limitations, likely exists. Studies reporting negative results or high complication rates may be underrepresented, potentially inflating apparent success rates (104). Single-center study predominance from high-volume academic centers may limit generalizability to community practice settings with different patient populations and resource availability.

### Surgery-first approaches and technological innovations

Surgery-first approach emergence represents a paradigm shift warranting discussion despite limited inclusion due to insufficient long-term data. Preliminary evidence suggests potential treatment duration and patient satisfaction advantages, though occlusal stability concerns and achieving precise post-surgical dental decompensation challenges require further investigation (105). Long-term comparative data (>5 years) lack limits definitive universal adoption recommendations.

Technological innovations including virtual surgical planning, 3D printing, and patient-specific surgical guides promise enhanced precision and outcomes (106). However, critical evaluation reveals limited high-quality evidence demonstrating patient-centered outcome superiority over conventional techniques. While accuracy improvements are documented, their translation to better clinical outcomes, quality of life, or complication reduction remains inadequately demonstrated. Cost-effectiveness analyses are notably absent, limiting universal adoption recommendations in resource-constrained settings.

### Psychological and psychosocial considerations

While quality of life improvements are well-documented, deeper psychological outcomes receive less attention. Studies specifically examining body image, self-esteem, and psychological disorders (depression, anxiety) remain limited. The finding that mental component scores improve more than physical component scores suggests important psychological benefits, but dedicated psychiatric assessments are rare (107). Furthermore, a subset of patients (5%–10%) report disappointment despite technically successful outcomes, highlighting expectation management complexity and enhanced preoperative psychological screening needs (108).

The relationship between objective skeletal changes and subjective patient satisfaction is not uniformly linear, with some studies documenting dissatisfaction despite excellent cephalometric outcomes. This disconnect underscores that technical success does not guarantee patient satisfaction and emphasizes comprehensive preoperative assessment importance including psychological evaluation, realistic expectation setting, and patient-centered goal definition (109).

### Implications for clinical practice

Several recommendations for clinical practice emerge from this systematic review:

1. Patient Selection and Counseling: Comprehensive preoperative assessment should include skeletal and dental evaluation, psychological screening, and quality of life assessment. Risk factor identification (age, advancement magnitude, skeletal pattern) should inform personalized complication risk estimates rather than pooled averages (110).
2. Informed Consent: Disclosure should include realistic complication rates, distinguishing temporary from permanent sequelae. Neurosensory disturbances should be highlighted given high incidence, though favorable recovery trajectory should be emphasized. Relapse risk and possible additional procedures should be explicitly discussed.
3. Outcome Assessment: Routine incorporation of validated QoL instruments at standardized timepoints (preoperative, 6, 12, 24 months) should become standard practice. Both clinician-reported and patient-reported outcomes are important and may diverge.
4. Long-term Follow-up: Enhanced surveillance for late complications, particularly progressive condylar resorption and TMJ disorders, is warranted. Follow-up protocols extending beyond conventional 12–24 months should be considered for high-risk patients.

### Limitations of this review

This systematic review has several limitations. First, Restriction to English-language studies may introduce language bias. Second, substantial heterogeneity across studies limited quantitative synthesis for several outcomes, necessitating narrative synthesis more susceptible to subjective interpretation. Third, evidence quality, as reflected in GRADE assessments, ranged from low to high, with many outcomes rated moderate certainty due to bias risk and inconsistency.

Fourth, individual patient data lack precluded more sophisticated analyses exploring patient characteristic, surgical technique, and outcome interactions. Fifth, publication bias assessment was limited by challenges applying standard methods to heterogeneous observational studies. Finally, quantitative outcome focus may underemphasize important qualitative patient experience aspects that questionnaires incompletely capture.

### Future research directions

This review identifies several critical research priorities:

1. Standardization Initiatives: Core outcome set development and validation for orthognathic surgery, including consensus on outcome definitions, measurement instruments, and assessment timepoints.[103]
2. Long-term Outcomes: Prospective studies with extended follow-up (≥5 years) capturing late complications, skeletal stability, and sustained quality of life improvements. Particular attention to progressive condylar resorption and late TMJ complications is needed.
3. Predictive Modeling: Validated prediction model development incorporating multiple variables to estimate individual patient complication risks and outcome probabilities, potentially incorporating genetic, biomechanical, and psychosocial factors.
4. Comparative Effectiveness Research: High-quality studies comparing surgery-first vs. conventional approaches, different fixation methods, and novel technologies with adequate power and patient-centered outcomes.
5. Economic Evaluation: Comprehensive cost-effectiveness analyses comparing orthognathic surgery to alternative management strategies and examining technological innovation cost-effectiveness.
6. Psychological Outcomes: Studies specifically examining psychological outcomes, expectation management, and factors predicting patient satisfaction beyond technical surgical success.

## Conclusion

This systematic review provides comprehensive synthesis of contemporary evidence regarding orthognathic surgery outcomes, encompassing 65 studies and 6,482 patients. The findings demonstrate that orthognathic surgery effectively corrects dentofacial deformities with significant improvements in skeletal relationships, dental occlusion, and functional parameters. Clinical outcomes show mean ANB angle improvements of 6.8° for class III and 5.4° for class II corrections, with skeletal stability maintained in the majority of patients. However, relapse occurs in approximately 19% of cases, with risk factors including advancement magnitude exceeding 7 mm and specific movement patterns.

The complication profile reveals an overall rate of 32.4%, predominantly consisting of minor, self-limiting events. Neurosensory disturbances represent the most frequent complication, affecting nearly half of patients undergoing bilateral sagittal split osteotomy, though permanent alterations occur in only 3.4%. Major complications requiring intervention remain relatively uncommon (4.2%), suggesting an acceptable safety profile for this elective procedure.

Quality of life improvements are substantial and sustained, with large effect sizes across multiple validated instruments. The greatest improvements occur in facial aesthetics and social functioning domains, with progressive enhancement reaching plateau by 12 months post-surgery. Patient satisfaction rates approximate 88%, though critical consideration reveals variability related to complications, expectation management, and baseline severity.

The evidence base, while substantial, is limited by methodological heterogeneity, predominance of observational designs, variable outcome definitions, and insufficient long-term follow-up. The lack of standardized core outcome sets complicates evidence synthesis and hinders robust clinical guideline development. Future research should prioritize standardization initiatives, long-term outcome studies, predictive modeling, and economic evaluation to advance evidence-based practice.

For clinical practice, these findings support orthognathic surgery as an effective treatment for dentofacial deformities when performed with appropriate patient selection, comprehensive preoperative assessment including psychological evaluation, and realistic expectation setting. Risk stratification based on identified factors should inform personalized counseling, and routine incorporation of patient-reported outcome measures should become standard practice. Enhanced surveillance for late complications, particularly in high-risk patients, warrants consideration.

In conclusion, orthognathic surgery achieves significant improvements in both objective clinical parameters and subjective quality of life for the majority of appropriately selected patients. However, the considerable complication rate and relapse risk necessitate careful patient selection, expert surgical technique, and comprehensive informed consent. Continued research addressing identified evidence gaps will further refine patient selection criteria and optimize treatment protocols to enhance outcomes and minimize complications.

## Data Availability

The original contributions presented in the study are included in the article/Supplementary Material, further inquiries can be directed to the corresponding author.
