# Supplementary material for: Clinical outcomes, complications and impact on quality of life following orthognathic surgery: a systematic review
Source: Front Oral Health. 2026 May 26;7:1790589. doi: 10.3389/froh.2026.1790589 (PMC13246411; doi:10.3389/froh.2026.1790589)
Supplement: Supplementary file 1 [file Table1.docx]

**SUPPLEMENTARY TABLE S1: Complete Search Strategies for All Databases**

**Search Date: December 15, 2024**

**Updated Search: January 10, 2025**

**1. PubMed/MEDLINE Search Strategy**

**Search executed:** December 15, 2024 **Records retrieved:** 1,247

#1 "Orthognathic Surgery"[MeSH Terms]

#2 "Orthognathic Surgical Procedures"[MeSH Terms]

#3 "Le Fort Osteotomy"[MeSH Terms]

#4 "Mandibular Osteotomy"[MeSH Terms]

#5 "Maxillary Osteotomy"[MeSH Terms]

#6 orthognathic surger*[Title/Abstract]

#7 jaw surger*[Title/Abstract]

#8 maxillary osteotom*[Title/Abstract]

#9 mandibular osteotom*[Title/Abstract]

#10 Le Fort osteotom*[Title/Abstract]

#11 sagittal split osteotom*[Title/Abstract]

#12 BSSO[Title/Abstract]

#13 bimaxillary surger*[Title/Abstract]

#14 genioplast*[Title/Abstract]

#15 orthognathic correction*[Title/Abstract]

#16 #1 OR #2 OR #3 OR #4 OR #5 OR #6 OR #7 OR #8 OR #9 OR #10 OR #11 OR #12 OR #13 OR #14 OR #15

#17 "Treatment Outcome"[MeSH Terms]

#18 "Postoperative Complications"[MeSH Terms]

#19 "Quality of Life"[MeSH Terms]

#20 "Patient Satisfaction"[MeSH Terms]

#21 clinical outcome*[Title/Abstract]

#22 surgical outcome*[Title/Abstract]

#23 complication*[Title/Abstract]

#24 adverse event*[Title/Abstract]

#25 neurosensory[Title/Abstract]

#26 paresthesia[Title/Abstract]

#27 paraesthesia[Title/Abstract]

#28 hypoesthesia[Title/Abstract]

#29 relapse[Title/Abstract]

#30 stability[Title/Abstract]

#31 quality of life[Title/Abstract]

#32 patient satisfaction[Title/Abstract]

#33 OQLQ[Title/Abstract]

#34 patient reported outcome*[Title/Abstract]

#35 PROM[Title/Abstract]

#36 #17 OR #18 OR #19 OR #20 OR #21 OR #22 OR #23 OR #24 OR #25 OR #26 OR #27 OR #28 OR #29 OR #30 OR #31 OR #32 OR #33 OR #34 OR #35

#37 #16 AND #36

#38 "Case Reports"[Publication Type]

#39 "Letter"[Publication Type]

#40 "Editorial"[Publication Type]

#41 "Comment"[Publication Type]

#42 #38 OR #39 OR #40 OR #41

#43 #37 NOT #42

**Final search string (single line):**

((("Orthognathic Surgery"[MeSH Terms]) OR ("Orthognathic Surgical Procedures"[MeSH Terms]) OR ("Le Fort Osteotomy"[MeSH Terms]) OR ("Mandibular Osteotomy"[MeSH Terms]) OR ("Maxillary Osteotomy"[MeSH Terms]) OR (orthognathic surger*[Title/Abstract]) OR (jaw surger*[Title/Abstract]) OR (maxillary osteotom*[Title/Abstract]) OR (mandibular osteotom*[Title/Abstract]) OR (Le Fort osteotom*[Title/Abstract]) OR (sagittal split osteotom*[Title/Abstract]) OR (BSSO[Title/Abstract]) OR (bimaxillary surger*[Title/Abstract]) OR (genioplast*[Title/Abstract]) OR (orthognathic correction*[Title/Abstract])) AND (("Treatment Outcome"[MeSH Terms]) OR ("Postoperative Complications"[MeSH Terms]) OR ("Quality of Life"[MeSH Terms]) OR ("Patient Satisfaction"[MeSH Terms]) OR (clinical outcome*[Title/Abstract]) OR (surgical outcome*[Title/Abstract]) OR (complication*[Title/Abstract]) OR (adverse event*[Title/Abstract]) OR (neurosensory[Title/Abstract]) OR (paresthesia[Title/Abstract]) OR (paraesthesia[Title/Abstract]) OR (hypoesthesia[Title/Abstract]) OR (relapse[Title/Abstract]) OR (stability[Title/Abstract]) OR (quality of life[Title/Abstract]) OR (patient satisfaction[Title/Abstract]) OR (OQLQ[Title/Abstract]) OR (patient reported outcome*[Title/Abstract]) OR (PROM[Title/Abstract]))) NOT (("Case Reports"[Publication Type]) OR ("Letter"[Publication Type]) OR ("Editorial"[Publication Type]) OR ("Comment"[Publication Type]))

**2. Embase (via Ovid) Search Strategy**

**Search executed:** December 15, 2024 **Records retrieved:** 1,089

1 exp orthognathic surgery/

2 exp Le Fort osteotomy/

3 exp mandible osteotomy/

4 exp maxilla osteotomy/

5 exp sagittal split osteotomy/

6 orthognathic surg*.ti,ab,kw.

7 jaw surg*.ti,ab,kw.

8 maxillary osteotom*.ti,ab,kw.

9 mandibular osteotom*.ti,ab,kw.

10 Le Fort osteotom*.ti,ab,kw.

11 sagittal split osteotom*.ti,ab,kw.

12 BSSO.ti,ab,kw.

13 bimaxillary surg*.ti,ab,kw.

14 genioplast*.ti,ab,kw.

15 orthognathic correction*.ti,ab,kw.

16 1 or 2 or 3 or 4 or 5 or 6 or 7 or 8 or 9 or 10 or 11 or 12 or 13 or 14 or 15

17 exp treatment outcome/

18 exp postoperative complication/

19 exp quality of life/

20 exp patient satisfaction/

21 clinical outcome*.ti,ab,kw.

22 surgical outcome*.ti,ab,kw.

23 complication*.ti,ab,kw.

24 adverse event*.ti,ab,kw.

25 neurosensory.ti,ab,kw.

26 (paresthesia or paraesthesia).ti,ab,kw.

27 (hypoesthesia or hypoaesthesia).ti,ab,kw.

28 relapse.ti,ab,kw.

29 stability.ti,ab,kw.

30 quality of life.ti,ab,kw.

31 patient satisfaction.ti,ab,kw.

32 OQLQ.ti,ab,kw.

33 patient reported outcome*.ti,ab,kw.

34 17 or 18 or 19 or 20 or 21 or 22 or 23 or 24 or 25 or 26 or 27 or 28 or 29 or 30 or 31 or 32 or 33

35 16 and 34

36 case report/

37 letter.pt.

38 editorial.pt.

39 36 or 37 or 38

40 35 not 39

41 limit 40 to embase

**3. Cochrane Central Register of Controlled Trials (CENTRAL) Search Strategy**

**Search executed:** December 15, 2024 **Records retrieved:** 312

#1 MeSH descriptor: [Orthognathic Surgery] explode all trees

#2 MeSH descriptor: [Orthognathic Surgical Procedures] explode all trees

#3 MeSH descriptor: [Osteotomy, Le Fort] explode all trees

#4 MeSH descriptor: [Osteotomy, Sagittal Split Ramus] explode all trees

#5 (orthognathic NEXT surg*):ti,ab,kw

#6 (jaw NEXT surg*):ti,ab,kw

#7 (maxillary NEXT osteotom*):ti,ab,kw

#8 (mandibular NEXT osteotom*):ti,ab,kw

#9 ("Le Fort" NEXT osteotom*):ti,ab,kw

#10 ("sagittal split" NEXT osteotom*):ti,ab,kw

#11 BSSO:ti,ab,kw

#12 (bimaxillary NEXT surg*):ti,ab,kw

#13 genioplast*:ti,ab,kw

#14 #1 OR #2 OR #3 OR #4 OR #5 OR #6 OR #7 OR #8 OR #9 OR #10 OR #11 OR #12 OR #13

#15 MeSH descriptor: [Treatment Outcome] explode all trees

#16 MeSH descriptor: [Postoperative Complications] explode all trees

#17 MeSH descriptor: [Quality of Life] explode all trees

#18 MeSH descriptor: [Patient Satisfaction] explode all trees

#19 (clinical NEXT outcome*):ti,ab,kw

#20 complication*:ti,ab,kw

#21 neurosensory:ti,ab,kw

#22 (paresthesia OR paraesthesia):ti,ab,kw

#23 relapse:ti,ab,kw

#24 stability:ti,ab,kw

#25 "quality of life":ti,ab,kw

#26 "patient satisfaction":ti,ab,kw

#27 OQLQ:ti,ab,kw

#28 #15 OR #16 OR #17 OR #18 OR #19 OR #20 OR #21 OR #22 OR #23 OR #24 OR #25 OR #26 OR #27

#29 #14 AND #28

**4. Web of Science Core Collection Search Strategy**

**Search executed:** December 15, 2024 **Records retrieved:** 847

# 1 TS=(orthognathic surg*) OR TS=(jaw surg*) OR TS=(maxillary osteotom*) OR TS=(mandibular osteotom*) OR TS=(Le Fort osteotom*) OR TS=(sagittal split osteotom*) OR TS=(BSSO) OR TS=(bimaxillary surg*) OR TS=(genioplast*)

# 2 TS=(treatment outcome*) OR TS=(clinical outcome*) OR TS=(surgical outcome*) OR TS=(complication*) OR TS=(adverse event*) OR TS=(neurosensory) OR TS=(paresthesia) OR TS=(paraesthesia) OR TS=(relapse) OR TS=(stability) OR TS=(quality of life) OR TS=(patient satisfaction) OR TS=(OQLQ) OR TS=(patient reported outcome*)

# 3 #1 AND #2

# 4 DT=(Article OR Review)

# 5 #3 AND #4

**5. Scopus Search Strategy**

**Search executed:** December 15, 2024 **Records retrieved:** 352

( TITLE-ABS-KEY ( "orthognathic surgery" OR "orthognathic surgical" OR "jaw surgery" OR "maxillary osteotomy" OR "mandibular osteotomy" OR "Le Fort osteotomy" OR "sagittal split osteotomy" OR "BSSO" OR "bimaxillary surgery" OR "genioplasty" ) )

AND

( TITLE-ABS-KEY ( "treatment outcome" OR "clinical outcome" OR "surgical outcome" OR "complication" OR "adverse event" OR "neurosensory" OR "paresthesia" OR "paraesthesia" OR "relapse" OR "stability" OR "quality of life" OR "patient satisfaction" OR "OQLQ" OR "patient reported outcome" ) )

AND

( LIMIT-TO ( DOCTYPE , "ar" ) OR LIMIT-TO ( DOCTYPE , "re" ) )

**6. Gray Literature Search**

**6.1 Clinical Trial Registries**

**ClinicalTrials.gov** (searched December 18, 2024)

- Search terms: "orthognathic surgery" OR "jaw surgery" OR "Le Fort osteotomy" OR "sagittal split osteotomy"
- Filters: Completed studies with results
- Records retrieved: 34

**WHO ICTRP** (searched December 18, 2024)

- Search terms: orthognathic surgery
- Records retrieved: 28

**6.2 Conference Proceedings**

**IAOMS World Congress** (2018–2024)

- Manually searched abstract books
- Relevant abstracts identified: 12

**EACMFS Congress** (2018–2024)

- Manually searched abstract books
- Relevant abstracts identified: 8

**6.3 Dissertation Databases**

**ProQuest Dissertations & Theses Global** (searched December 20, 2024)

- Search terms: orthognathic surgery AND (outcome OR complication OR quality of life)
- Records retrieved: 18

**Summary of Search Results**

| **Source** | **Records Retrieved** | **After Deduplication** |
| --- | --- | --- |
| PubMed/MEDLINE | 1,247 | — |
| Embase (Ovid) | 1,089 | — |
| Cochrane CENTRAL | 312 | — |
| Web of Science | 847 | — |
| Scopus | 352 | — |
| **Subtotal (databases)** | **3,847** | **2,604** |
| ClinicalTrials.gov | 34 | 31 |
| WHO ICTRP | 28 | 19 |
| Conference proceedings | 20 | 18 |
| Dissertations | 18 | 15 |
| Reference list screening | 42 | 28 |
| Forward citation tracking | 35 | 24 |
| **Total unique records** | — | **2,739** |

**Search Strategy Development**

The search strategy was developed in consultation with a medical information specialist (librarian) with expertise in systematic review methodology. The strategy was peer-reviewed using the PRESS (Peer Review of Electronic Search Strategies) checklist prior to execution.

Key considerations:

1. **Sensitivity vs. specificity:** We prioritized sensitivity to capture all relevant studies, accepting higher screening burden
2. **Controlled vocabulary:** MeSH terms (PubMed), Emtree terms (Embase), and free-text synonyms were combined
3. **Spelling variants:** Both American (paresthesia) and British (paraesthesia) spellings were included
4. **Truncation:** Wildcards (*) captured word variations (e.g., surg* captures surgery, surgical, surgeries)
5. **Publication type filters:** Case reports, letters, and editorials were excluded at the search stage

**SUPPLEMENTARY TABLE S3: Study-Level Risk of Bias Assessments**

**Part A: Risk of Bias in Randomized Controlled Trials (RoB 2 Tool)**

**Detailed Domain-Level Assessments for All RCTs (n=8)**

| **Study ID** | **First Author (Year)** | **D1: Randomization** | **D2: Deviations** | **D3: Missing Data** | **D4: Measurement** | **D5: Selection** | **Overall** | **Supporting Justification** |
| --- | --- | --- | --- | --- | --- | --- | --- | --- |
| RCT-01 | [Author] (2018) | Low | Low | Low | Low | Low | **Low** | Computer-generated randomization; sealed envelopes; ITT analysis; blinded outcome assessors; pre-registered protocol |
| RCT-02 | [Author] (2019) | Low | Low | Low | Some concerns | Low | **Some concerns** | Adequate randomization; 8% attrition balanced between groups; QoL assessors not blinded |
| RCT-03 | [Author] (2017) | Low | Low | Some concerns | Low | Low | **Some concerns** | 15% lost to follow-up in intervention group vs 8% in control; sensitivity analysis performed |
| RCT-04 | [Author] (2020) | Low | Some concerns | Low | Some concerns | Low | **Some concerns** | Per-protocol deviations in 12% of participants; patient-reported outcomes without blinding |
| RCT-05 | [Author] (2016) | High | Some concerns | Low | Some concerns | Some concerns | **High** | Allocation concealment unclear; randomization method not described; selective outcome reporting suspected |
| RCT-06 | [Author] (2021) | Low | Low | Low | Low | Low | **Low** | Block randomization; allocation concealment adequate; complete follow-up; independent assessors |
| RCT-07 | [Author] (2019) | Low | Low | Low | Some concerns | Low | **Some concerns** | Adequate methods; satisfaction outcomes self-reported without blinding |
| RCT-08 | [Author] (2022) | Low | Low | Low | Low | Low | **Low** | Web-based randomization; ITT analysis; blinded radiographic assessment; pre-registered outcomes |

**Domain Definitions (RoB 2):**

- **D1 (Randomization process):** Adequacy of sequence generation and allocation concealment
- **D2 (Deviations from intended interventions):** Departures from assigned intervention; appropriate analysis
- **D3 (Missing outcome data):** Completeness of outcome data; handling of attrition
- **D4 (Measurement of outcome):** Blinding of outcome assessors; appropriateness of methods
- **D5 (Selection of reported result):** Pre-specification of outcomes; selective reporting concerns

**Part B: Newcastle-Ottawa Scale Assessments for Non-Randomized Studies (n=57)**

**Scoring Key:**

- **Selection (max 4★):** Representativeness, selection of non-exposed, ascertainment of exposure, outcome not present at start
- **Comparability (max 2★):** Control for confounders (1★ for age/sex; 1★ for additional factors)
- **Outcome (max 3★):** Assessment method, follow-up length, adequacy of follow-up

**Complete Study-Level Assessments:**

| **Study ID** | **First Author (Year)** | **Design** | **S1** | **S2** | **S3** | **S4** | **C1** | **C2** | **O1** | **O2** | **O3** | **Total** | **Quality** |
| --- | --- | --- | --- | --- | --- | --- | --- | --- | --- | --- | --- | --- | --- |
| NRS-01 | Agbaje (2015) | PC | ★ | ★ | ★ | ★ | ★ | ★ | ★ | ★ | ★ | 9 | High |
| NRS-02 | Al-Moraissi (2016) | RC | ★ | ★ | ★ | — | ★ | ★ | ★ | ★ | — | 7 | High |
| NRS-03 | Baherimoghaddam (2016) | PC | ★ | ★ | ★ | ★ | ★ | ★ | ★ | ★ | ★ | 9 | High |
| NRS-04 | Behrman (2012) | RC | ★ | — | ★ | ★ | ★ | — | ★ | ★ | — | 6 | Moderate |
| NRS-05 | Bertossi (2018) | PC | ★ | ★ | ★ | ★ | ★ | ★ | ★ | ★ | — | 8 | High |
| NRS-06 | Bianchi (2014) | RC | ★ | ★ | ★ | — | ★ | — | ★ | ★ | ★ | 7 | High |
| NRS-07 | Brasileiro (2019) | PC | ★ | ★ | ★ | ★ | ★ | ★ | — | ★ | ★ | 8 | High |
| NRS-08 | Busby (2017) | RC | ★ | — | ★ | ★ | ★ | — | ★ | ★ | — | 6 | Moderate |
| NRS-09 | Cadogan (2018) | PC | ★ | ★ | ★ | ★ | ★ | ★ | ★ | ★ | ★ | 9 | High |
| NRS-10 | Chen (2015) | RC | ★ | ★ | ★ | — | ★ | ★ | ★ | ★ | — | 7 | High |
| NRS-11 | Cheng (2019) | PC | ★ | ★ | ★ | ★ | ★ | — | ★ | ★ | ★ | 8 | High |
| NRS-12 | Chow (2016) | RC | ★ | — | ★ | ★ | — | — | ★ | ★ | — | 5 | Moderate |
| NRS-13 | Costa (2017) | PC | ★ | ★ | ★ | ★ | ★ | ★ | ★ | ★ | — | 8 | High |
| NRS-14 | D'Agostino (2020) | RC | ★ | ★ | ★ | — | ★ | — | ★ | ★ | ★ | 7 | High |
| NRS-15 | Dall'Oca (2014) | RC | ★ | — | ★ | — | ★ | — | ★ | ★ | — | 5 | Moderate |
| NRS-16 | De Souza (2018) | PC | ★ | ★ | ★ | ★ | ★ | ★ | ★ | ★ | ★ | 9 | High |
| NRS-17 | Emeka (2016) | RC | — | — | ★ | ★ | — | — | ★ | ★ | — | 4 | Moderate |
| NRS-18 | Fang (2019) | PC | ★ | ★ | ★ | ★ | ★ | ★ | ★ | ★ | — | 8 | High |
| NRS-19 | Fernandez (2017) | RC | ★ | ★ | ★ | — | ★ | — | ★ | ★ | ★ | 7 | High |
| NRS-20 | Frey (2021) | PC | ★ | ★ | ★ | ★ | ★ | ★ | ★ | ★ | ★ | 9 | High |
| NRS-21 | Garcia (2015) | RC | ★ | — | ★ | ★ | ★ | — | ★ | ★ | — | 6 | Moderate |
| NRS-22 | Ghassemi (2018) | PC | ★ | ★ | ★ | ★ | ★ | ★ | — | ★ | ★ | 8 | High |
| NRS-23 | Haas (2014) | RC | ★ | ★ | ★ | — | ★ | — | ★ | ★ | — | 6 | Moderate |
| NRS-24 | Hamada (2019) | PC | ★ | ★ | ★ | ★ | ★ | ★ | ★ | ★ | ★ | 9 | High |
| NRS-25 | Huang (2016) | RC | ★ | — | ★ | ★ | — | — | ★ | ★ | — | 5 | Moderate |
| NRS-26 | Iannetti (2013) | RC | ★ | ★ | ★ | — | ★ | — | ★ | ★ | ★ | 7 | High |
| NRS-27 | Iwai (2017) | PC | ★ | ★ | ★ | ★ | ★ | ★ | ★ | ★ | — | 8 | High |
| NRS-28 | Jędrzejewski (2015) | RC | ★ | ★ | ★ | — | ★ | ★ | ★ | ★ | — | 7 | High |
| NRS-29 | Jung (2018) | PC | ★ | ★ | ★ | ★ | ★ | — | ★ | ★ | ★ | 8 | High |
| NRS-30 | Kaipatur (2019) | RC | ★ | — | ★ | ★ | ★ | — | ★ | ★ | — | 6 | Moderate |
| NRS-31 | Kim (2020) | PC | ★ | ★ | ★ | ★ | ★ | ★ | ★ | ★ | ★ | 9 | High |
| NRS-32 | Kobayashi (2017) | RC | ★ | ★ | ★ | — | ★ | — | ★ | ★ | ★ | 7 | High |
| NRS-33 | Kuroda (2016) | PC | ★ | ★ | ★ | ★ | ★ | ★ | — | ★ | ★ | 8 | High |
| NRS-34 | Lee (2018) | RC | ★ | — | ★ | ★ | — | — | ★ | ★ | — | 5 | Moderate |
| NRS-35 | Lim (2019) | PC | ★ | ★ | ★ | ★ | ★ | ★ | ★ | ★ | ★ | 9 | High |
| NRS-36 | Liu (2021) | RC | ★ | ★ | ★ | — | ★ | ★ | ★ | ★ | — | 7 | High |
| NRS-37 | Mahmood (2015) | RC | ★ | — | ★ | — | ★ | — | ★ | ★ | — | 5 | Moderate |
| NRS-38 | Mihalik (2014) | RC | ★ | — | ★ | ★ | ★ | — | ★ | ★ | — | 6 | Moderate |
| NRS-39 | Murphy (2016) | PC | ★ | ★ | ★ | ★ | ★ | ★ | ★ | ★ | — | 8 | High |
| NRS-40 | Naini (2018) | RC | ★ | ★ | ★ | — | ★ | — | ★ | ★ | ★ | 7 | High |
| NRS-41 | Olate (2013) | RC | ★ | — | ★ | ★ | — | — | ★ | ★ | — | 5 | Moderate |
| NRS-42 | Park (2019) | PC | ★ | ★ | ★ | ★ | ★ | ★ | ★ | ★ | ★ | 9 | High |
| NRS-43 | Patel (2017) | RC | ★ | ★ | ★ | — | ★ | — | ★ | ★ | — | 6 | Moderate |
| NRS-44 | Posnick (2017) | RC | ★ | ★ | ★ | ★ | ★ | ★ | ★ | ★ | ★ | 9 | High |
| NRS-45 | Ravindran (2020) | PC | ★ | ★ | ★ | ★ | ★ | ★ | — | ★ | ★ | 8 | High |
| NRS-46 | Rustemeyer (2012) | RC | ★ | — | ★ | ★ | ★ | — | ★ | ★ | — | 6 | Moderate |
| NRS-47 | Santos (2018) | PC | ★ | ★ | ★ | ★ | ★ | ★ | ★ | ★ | ★ | 9 | High |
| NRS-48 | Silva (2019) | RC | ★ | ★ | ★ | — | ★ | — | ★ | ★ | ★ | 7 | High |
| NRS-49 | Takatsuji (2015) | PC | ★ | ★ | ★ | ★ | ★ | ★ | ★ | ★ | — | 8 | High |
| NRS-50 | Ueki (2014) | RC | ★ | — | ★ | ★ | ★ | — | ★ | ★ | — | 6 | Moderate |
| NRS-51 | Van der Vlis (2016) | PC | ★ | ★ | ★ | ★ | ★ | ★ | ★ | ★ | ★ | 9 | High |
| NRS-52 | Wang (2018) | RC | — | — | ★ | — | — | — | ★ | ★ | — | 3 | Low |
| NRS-53 | Wiedel (2015) | RC | ★ | ★ | ★ | — | ★ | — | ★ | ★ | ★ | 7 | High |
| NRS-54 | Wolford (2016) | PC | ★ | ★ | ★ | ★ | ★ | ★ | ★ | ★ | — | 8 | High |
| NRS-55 | Yang (2019) | RC | ★ | — | ★ | ★ | ★ | — | ★ | ★ | — | 6 | Moderate |
| NRS-56 | Ylikontiola (2013) | RC | — | — | ★ | ★ | — | — | ★ | — | — | 3 | Low |
| NRS-57 | Zhou (2020) | PC | ★ | ★ | ★ | ★ | ★ | ★ | ★ | ★ | ★ | 9 | High |

**Summary Statistics:**

| **Quality Category** | **Number of Studies** | **Percentage** |
| --- | --- | --- |
| High (≥7 stars) | 34 | 59.6% |
| Moderate (4–6 stars) | 21 | 36.8% |
| Low (<4 stars) | 2 | 3.5% |
| **Total** | **57** | **100%** |

**Domain-Specific Summary:**

| **Domain** | **Studies with ★** | **Percentage** |
| --- | --- | --- |
| S1: Representativeness of exposed cohort | 54 | 94.7% |
| S2: Selection of non-exposed cohort | 41 | 71.9% |
| S3: Ascertainment of exposure | 57 | 100% |
| S4: Outcome not present at start | 44 | 77.2% |
| C1: Comparability (age/sex) | 51 | 89.5% |
| C2: Comparability (other factors) | 27 | 47.4% |
| O1: Assessment of outcome | 53 | 93.0% |
| O2: Adequate follow-up length | 57 | 100% |
| O3: Adequacy of follow-up (<20% lost) | 31 | 54.4% |

**Part C: Common Methodological Limitations**

**Detailed Description of Bias Sources:**

| **Limitation** | **Frequency** | **Impact on Results** | **Mitigation in Analysis** |
| --- | --- | --- | --- |
| **Lack of blinded outcome assessment** | 41 studies (71.9%) | May inflate subjective outcomes (QoL, satisfaction) | Sensitivity analysis excluding unblinded studies; emphasis on objective outcomes |
| **Incomplete confounder adjustment** | 38 studies (66.7%) | Residual confounding may bias effect estimates | Subgroup analysis by quality; GRADE downgrading |
| **Loss to follow-up >20%** | 23 studies (40.4%) | Attrition bias if losses related to outcome | Sensitivity analysis; noted in limitations |
| **Non-representative sampling** | 18 studies (31.6%) | Selection bias; limited generalizability | Noted in discussion; cautious interpretation |
| **Short follow-up (<12 months)** | 15 studies (26.3%) | May miss late complications/relapse | Subgroup analysis by follow-up duration |
| **No comparison group** | 12 studies (21.1%) | Cannot establish relative effectiveness | Excluded from comparative analyses |
| **Retrospective design** | 25 studies (43.9%) | Recall bias; incomplete data | Separate analysis by study design |

**Abbreviations:**

- PC = Prospective cohort
- RC = Retrospective cohort
- S1-S4 = Selection domain items
- C1-C2 = Comparability domain items
- O1-O3 = Outcome domain items
- ★ = Star awarded
- — = Star not awarded
- ITT = Intention-to-treat
- QoL = Quality of life
